# Profiling the host response to malaria vaccination and malaria challenge

**DOI:** 10.1016/j.vaccine.2015.07.107

**Published:** 2015-09-29

**Authors:** Susanna Dunachie, Adrian V.S. Hill, Helen A. Fletcher

**Affiliations:** a The Jenner Institute, Nuffield Department of Medicine, University of Oxford, Churchill Hospital, Oxford OX3 7LJ, UK; b Mahidol-Oxford Tropical Medicine Research Unit, 3rd Floor, 60th Anniversary Chalermprakiat Building, 420/6 Ratchawithi Road, Bangkok 10400, Thailand; c Centre for Tropical Medicine and Global Health, Nuffield Department of Medicine Research Building, University of Oxford, Old Road Campus, Roosevelt Drive, Oxford OX3 7FZ, UK; d London School of Hygiene & Tropical Medicine, London, W1CE 7HT, UK

**Keywords:** *Plasmodium falciparum*, Malaria, Vaccine, Microarray, Biomarkers, Transcript, Immunity

## Abstract

A vaccine for malaria is urgently required. The RTS,S vaccine represents major progress, but is only partially effective. Development of the next generation of highly effective vaccines requires elucidation of the protective immune response. Immunity to malaria is known to be complex, and pattern-based approaches such as global gene expression profiling are ideal for understanding response to vaccination and protection against disease. The availability of experimental sporozoite challenge in humans to test candidate malaria vaccines offers a precious opportunity unavailable for other current targets of vaccine research such as HIV, tuberculosis and Ebola. However, a limited number of transcriptional profiling studies in the context of malaria vaccine research have been published to date. This review outlines the background, existing studies, limits and opportunities for gene expression studies to accelerate malaria vaccine research.

## 1 Introduction

Malaria remains one of the world's biggest killers with an estimated 584,000 deaths attributable to *Plasmodium falciparum* infection in 2013 [1]. The spectres of emerging resistance of the parasite to artemisinin drugs [2], and increasing resistance of mosquitoes to insecticides [3] mean a vaccine is urgently required. There has been considerable progress towards a vaccine, most notably the RTS,S vaccine, but this vaccine is only partially effective [4]. In order to design the next generation of vaccines it is imperative to maximise our understanding of the mechanisms of protective immune responses evoked by existing candidate vaccines. Malaria is unusual in having the advantage of human sporozoite challenge for rapid assessment of candidate vaccines. Exploration of mechanisms of immune protection is therefore valuable for vaccine research beyond malaria.

Naturally acquired immunity to malaria requires repeated exposure and is non-sterile, short-lived, and species-, strain- and variant-specific [5,6]. Protective immune responses against malaria may be humoral or cellular, and can be directed at the pre-erythrocytic parasite, the blood stage merozoite, or to malarial antigens on the surface of infected erythrocytes. The relative importance of different immune responses to malaria measured in exposed populations is unknown. Furthermore, many findings have not been reproduced in separate populations. It is likely that high level protection against malaria in humans depends on the summed effect of multiple low level immune responses to several antigens, with a different “protective signature” for each person depending on their genetic background [7].

Parasite diversity represents a major obstacle to malaria vaccine development [8]. For example, around 60 different *var* genes encode *P. falciparum* erythrocyte membrane protein 1 (PfEMP1) [9], and this parasite diversity contributes to the slow speed of acquisition of natural immunity [10]. Assessment of potential markers of immunity is hindered by the fact that specific responses may be short-lived and fluctuate with malaria season and degree of parasitaemia [11]. Although used extensively as surrogate markers of immunity to malaria for both vaccine studies and naturally exposed populations, neither antibody levels nor IFN-γ secretion, measured by ex vivo and cultured ELISPOT (enzyme-linked immunospot assay) correlate with protection consistently between studies. As malaria is an intracellular parasite, it is likely that a protective vaccine would activate the cellular arm of the immune response and be effective against the pre-erythrocytic liver stage of the life cycle. It is highly possible that a combination of several pathways is required. *P. falciparum* causes the majority of deaths, and this parasite is the focus of most research for a malaria vaccine. However, *Plasmodium vivax* represents a serious threat to global health.

Global pattern recognition approaches have great potential to unravel the mechanisms of protection against malaria by candidate malaria vaccines. Such approaches supplement traditional techniques of antibody measurement and quantification of specific pre-determined cellular responses. This review focuses on the use of transcriptomics in malaria vaccine research.

## 2 Malaria vaccines

The feasibility of a malaria vaccine is supported by two main findings. Firstly, people in endemic areas accumulate considerable protection against clinical disease [12]. Secondly, 90% of volunteers receiving a repeated series of irradiated sporozoites via infected mosquitoes demonstrate sterile protection to over several months [13]. This irradiated sporozoite approach was originally considered only a research tool because of practical issues scaling for tropical settings. However there is now a focussed programme evaluating mass production which has reported success in malaria-naïve subjects using a stored preparation of irradiated sporozoites delivered by the intravenous route [14].

Candidate vaccine regimens currently undergoing clinical trials can be viewed online in the WHO's “rainbow tables” [15] and in recent reviews [16,17]. Malaria vaccine strategies have typically targeted one or a few antigens from one of the distinct life-cycle stages. This is in contrast to naturally acquired immunity which is likely to involve broad spectrum immunity to a range of antigens across life-cycle stages [6]. Analysis of the *P. falciparum* proteome revealed that the expression of key malarial antigens is often not as stage-specific as was traditionally thought [18]. Nevertheless, certain antigens predominate at each stage. Increasingly, research groups are combining antigens from more than one lifecycle stage to develop “multi-stage” vaccines, although maintaining potency in a combined antigen approach can be problematic [19,20].

Pre-erythrocytic vaccines act during the parasite's brief journey from the skin to the liver, or target infected hepatocytes. The most advanced candidate malaria vaccine is the CSP-based subunit vaccine RTS,S (GlaxoSmithKline Biologicals, Rixensart, Belgium/Walter Reed Army Institute of Research). This is a protein-in-adjuvant vaccine, which targets the pre-erythrocytic stage circumsporozoite protein. A large number of clinical studies have featured in its development (reviewed in Ref. [21]), including a study comparing the adjuvants AS01B and AS02A with saline in 102 malaria-naïve healthy adults in the USA [22]. This study is a rich resource for exploring why some subjects were protected by the vaccine and some were not. A Phase III trial showed efficacy against first episode of clinical malaria of 46% in children in the first 18 months [4] but efficacy in infants was less at 27%. Efforts continue to improve this efficacy, building on previous attempts to combine RTS,S with other antigens [23] or combine the RTS,S vaccine in a prime-boost strategy [24,25].

Other pre-erythrocytic stage approaches include prime-boost strategies to deliver the same antigen in different carriers, often using viral vectors, to optimise the innate immune milieu for antigen presentation [26]. Examples include using simian adenovirus ChAd63 as a viral vector with modified vaccinia virus Ankara (MVA) encoding TRAP (thrombospondin-related adhesion protein) [27,28], and a DNA prime, human serotype 5 adenovirus boost regimen encoding both CSP and apical membrane antigen-1 (AMA-1) [29].

Blood stage vaccines are “anti-disease” vaccines that aim to prevent invasion of blood cells after the liver stage, or prevent the complications of disease, by targeting merozoite invasion of erythrocytes, parasitised erythrocytes or prevent sequestration. Finally, transmission-blocking vaccines seek to generate immunity against sexual stages of the parasite [30,31]. However, current published studies available for transcriptome studies in malaria vaccine research are for pre-erythrocytic vaccines, which are the focus of this review.

## 3 Controlled human malaria infection studies

The availability of a human experimental sporozoite challenge model for assessing protective efficacy of candidate vaccines provides an excellent opportunity to study immune responses following vaccination in relation to efficacy (reviewed in Refs. [32,33]. Vaccinated volunteers and control participants undergo controlled human malaria infection (CHMI) challenge with *P. falciparum* at the peak vaccine immune response by one of three methods: via the bites of infectious mosquitoes [34,35], by needle-based administration of cryopreserved sporozoites [36] or by infected red blood cells [37,38]. For the exposure to mosquito bites CHMI method, laboratory-reared *Anopheles stephensi* mosquitoes are infected with drug-sensitive 3D7 or NF54 strain *P. falciparum* parasites by gametocyte culture and feeding [39]. Each participant is then exposed to the bites of five infected mosquitoes. Subjects are monitored intensively by blood film and quantitative PCR analysis, and treated immediately on reaching a challenge endpoint. Vaccinated subjects can be completely protected against challenge (“sterile protection”), show a delay to parasitaemia of at least 48 h compared to unvaccinated control subjects consistent with partial protection [40], or show no protection.

## 4 Immunity to malaria vaccines

There are many published studies exploring immune correlates of protection by the RTS,S vaccine. However, a large gap remains in our understanding of mechanisms of protection by pre-erythrocytic candidate vaccines. Anti-CSP antibodies are associated with protection, with a mathematical model predicting that a titre of 51 units/ml would prevent 50% of infections and clinical malaria episodes in children [41]. However, other immune mechanisms contributing to protection are not well characterised. Natural exposure does not induce strong levels of anti-CSP antibodies, and there is no evidence of boosting of RTS,S-induced anti-CSP responses by natural exposure [42]. A clinical study in malaria-naïve subjects comparing the two adjuvants AS02A and AS01B with saline [43] demonstrated significantly higher anti-CSP titres induced by both AS02A and AS01B adjuvants compared to saline, supporting the adjuvant being a crucial factor for antibody induction. A number of studies have explored T cell responses to RTS,S [43–49] with relationships described, including an independent association between CSP-specific TNFα+ CD4+ T cells and protection in Kenyan children [44]. Models support a contribution to protection by CD4+ T cells in malaria-naïve volunteers undergoing sporozoite challenge after RTS,S [50]. There is evidence from murine studies that anti-CSP specific CD8 T cells can protect against malaria [51] but RTS,S does not appear to be a potent induced of CD8 T cells. A greater understanding of the role of regulatory immune responses to candidate malaria vaccines is also required to optimise design [59]. Further work exploring the interaction in endemic regions between RTS,S vaccination, pre-existing immunity and post-vaccination malaria exposure is of importance. Interestingly a recent study of the breadth of humoral responses using protein arrays [52] was consistent with RTS,S vaccination leading to lower exposure to liver and blood stage parasites. This contrasted with the hypothesis that a partially effective RTS,S would lead to lower numbers of parasites emerging from the liver favouring a prolonged low level blood stage which in turn would facilitate naturally acquired blood stage immunity to supplement the anti-CSP antibodies. The protein array data supports an all-or-nothing hypothesis whereby malaria is either prevented by blocking sporozoite invasion of hepatocytes, or not.

The prime-boost approach is a good platform for induction of CD8 T cells [53,54]. A correlation in humans between IFN-γ-producing CD8+ T cells and protection against sporozoite challenge was reported for the ChAd63-MVA regimen encoding ME TRAP [27]. Elsewhere, analysis for immune correlates of protection in the four subjects protected by the DNA-Ad5 regimen encoding CSP AMA-1 [29] showed higher effector to central memory CD8+ T cell ratios to AMA1 in three subjects and to CSP in one subject [55].

## Transcriptional profiling of the vaccine response and integration with outcome data from clinical challenge studies

There are very limited studies available of transcriptional profiling in the context of malaria vaccine development. In a murine *Plasmodium yoelii* model, Tse et al. at Johns Hopkins University Bloomberg School of Public Health characterised the gene expression of CSP-specific memory CD8+ T cells residing in the liver and spleen after immunisation with irradiated sporozoites [56]. Differences in the expression of a number of genes involved in effector function, the cell cycle, cell trafficking, transcription and intracellular signalling were reported. There was evidence of persistent T-cell activation in the liver including upregulation of CD69 expression and genes from effector pathways, consistent with prolonged *Plasmodium* antigen presentation. However, the transcriptional profile in the liver did not resemble the “memory signature” seen by repeated *Listeria* exposure [57], or the T-cell exhaustion profile reported in chronic viral infection [58] in other murine studies. This study demonstrates the unique environment of the liver for the development of immunity to malaria, and is a reminder that studying transcription signature in only the peripheral blood is likely to miss some details of protective responses. The finding that the molecular signature for *Plasmodium*-exposed CD8+ T cells in the liver is distinct from those reported for other pathogens is important for understanding the mechanism of responses to malaria.

Vahey and colleagues at Walter Reed Army Institute of Research performed transcriptional profiling of 39 malaria-naïve human subjects in a study of RTS,S [59]. These subjects received either the AS01B or the AS02A adjuvant [22]. In a subsequent sporozoite challenge, 13/39 had sterile protection against malaria, 11/39 had a delay to parasitaemia and 15/39 had no protection. Gene expression studies were performed using peripheral blood mononuclear cells (PBMC) from subjects isolated at several points in the trial.

Transitory changes in genes relating to a number of inflammatory processes including apoptosis and protein kinase cascade were reported 24 h after the final vaccine. These could relate to the adjuvant, the Hepatitis B antigen in the vaccine or the CSP epitope. Five days after sporozoite challenge, principal component analysis (PCA) showed clustering of PBMC into distinct groups of those challenged but not vaccinated, those challenged and vaccinated, and the same subjects before vaccination or challenge. This demonstrated that subjects vaccinated with RTS,S respond differently to challenge compared to unvaccinated subjects, as early as five days post challenge, although the genes and pathways associated with these clusters were not reported. Classification and prediction analysis at this timepoint five days after challenge predicted with 100% accuracy whether a subject went on to be completely, partially or not protected against malaria, using a classifier set of 393 genes including genes associated with the cell cycle and regulation of apoptosis. This classifier set was only predictive when measured five days after challenge, and was unable to classify by outcome prior to challenge (two weeks after final vaccination). However gene set enrichment analysis (GSEA) at this pre-challenge timepoint found differential expression of genes in the proteasome degradation pathway in protected subjects compared to the non-protected subjects, in particular for genes in the “immunoproteasome” subgroup involved in processing peptides for Major Histocompatibility Complex (MHC) Class 1 pathways.

This work is important because RTS,S is not thought to be a potent inducer of CD8+ T cell responses, yet this study provides evidence of the pivotal role of CD8+/Class 1 pathways in defence against malaria in RTS,S vaccinated subjects. Achieving sufficient power to evaluate global gene expression two weeks after vaccination in the PBMC of humans who are not acutely unwell is a challenge because of noise from the heterogeneous genetic and environmental background of the subjects. Therefore, demonstration of differential expression here is a landmark step. The robustness of this approach is confirmed by a different study by an independent group [60] which sought to identify a common “vaccine transcriptomic signature” for responses to five different vaccines (two for *Neisseria meningitidis*, two for influenza and one for Yellow Fever). Of 1255 genes identified as a vaccine transcriptomic signature common to at least 4/5 of the vaccine datasets, 1231 (98%) were present in the dataset from the RTS,S study by Vahey et al. [59] when the RTS,S study was used as a test dataset.

A smaller study reported in this issue [61] evaluated response to vaccination in sixteen subjects receiving one of two prime-boost regimens: either RTS,S/AS02A and MVA-CS (CSP study), or DNA-ME TRAP and MVA-ME TRAP (TRAP study). 14/16 subjects underwent sporozoite challenge, and three subjects (two from CSP study and one from TRAP study) were completely protected against malaria with others showing a delay to parasitaemia. To focus on the antigen-specific response to vaccination, PBMC stimulated with peptides from the vaccines (either CSP or TRAP) were compared to paired unstimulated PBMC. To examine the transcription profile associated with protection against malaria, antigen-stimulated PBMC were normalised pairwise to matched unstimulated PBMC prior to analysis. The dominant transcriptional profile seen across the analyses was upregulation of genes found in the IFN-γ pathway. GSEA analysis revealed strong antigen-specific positive enrichment of genes associated with the proteasome after vaccination with ME-TRAP, which is consistent with the findings of Vahey and colleagues and may reflect the T-cell inducing capabilities of the prime-boost platform. Antigen-specific positive enrichment of genes associated with IFN induction and antigen presentation modules was seen in subjects with complete protection from malaria challenge. Antigen-specific negative enrichment of genes associated with stem cells, regulatory monocytes and myeloid modules was seen in protected subjects. This study confirms the benefit of focussing vaccine development efforts on strategies to optimise activation of the proteasome for efficient antigen presentation, and suggests approaches that favour a bias in haemopoietic precursor development towards lymphoid lineage may improve efficacy.

## 5 Limitations, opportunities and the future for transcriptomics

Transcriptional studies in vaccine research often use small sample sizes, suffer from limited immunogenicity of the vaccines being studied and represent a snapshot in time of gene expression. Confirmation in new datasets is required, and exploration beyond gene expression to demonstrate changes at the protein level is desirable. Further limits for these studies are the expense and expertise involved, but costs are decreasing and training in a systems-based analytic strategy can “democratize” the bioinformatics [62], improving accessibility to global multi-lingual researchers. PBMC are frequently cryopreserved in vaccine studies where cellular pathways are of interest as a means of sampling the current immune activity. However the study by Tse et al. [56] is a reminder of different expression occurring at different sites in the body, and animal models remain useful for allowing studies of sites inaccessible in human trials. The key role of neutrophils in response to intracellular pathogens is increasingly illuminated by transcriptional studies [63,64] and many researchers prefer to examine whole blood responses for this reason, although the development of approaches to examine cell subsets is of great interest [65].

The lack of clearly defined and consistent assays to predict protective immunity against malaria is a major obstacle in the development and testing of malaria vaccines. Elucidation of new correlates of protective immunity may indicate new targets for vaccination and allow efficient monitoring of population's response to vaccination. It is also an opportunity to learn more about antigen-specific immunity in general. Studies in malaria-naïve populations using irradiated sporozoites conferring high rates of protection [14] provide ideal opportunities to compare molecular signatures before and after immunisation and relate this to challenge, whilst trials of partially effective vaccines offer the chance to define the pattern of difference between protected and non-protected subjects. Anecdotally a number of researchers in this field have transcriptional profiling studies as part of their programme, and we hope that the next few years will herald more published reports.

Evaluation of response to candidate malaria vaccines in endemic populations is desirable. There are technical, financial and blood volume-limit difficulties in studying transcriptomics in the field, but the establishment of some flexibility in storage conditions for whole-blood in-tube RNA storage [66], and the development of solid RNA collection methods for use of dried blood spots [67] offer potential for tropical settings.

We live in an exciting time for the deployment of state-of-the-art tools to reveal the secrets of immunity to complex pathogens. Future approaches to understanding the mechanisms of protection of partially effective vaccines such as RTS,S should include a range of approaches alongside transcriptomics such as multiplex cytometry, protein arrays [68] and mass spectrometry. Transcriptomics is broadening our understanding of malaria vaccine induced protective immunity, and deeper integration of transcriptomics in clinical malaria vaccine trials will help to identify immune correlates and guide the design of future malaria vaccines.

## Conflict of interest

None declared.
